# Development of Chitosan/Poly(Vinyl Alcohol) Electrospun Nanofibers for Infection Related Wound Healing

**DOI:** 10.3389/fphys.2016.00683

**Published:** 2017-01-11

**Authors:** Mian Wang, Amit K. Roy, Thomas J. Webster

**Affiliations:** ^1^Department of Chemical Engineering, Northeastern UniversityBoston, MA, USA; ^2^Wenzhou Institute of Biomaterials and Engineering, Wenzhou Medical UniversityWenzhou, China; ^3^Center of Excellence for Advanced Materials Research, King Abdulaziz UniversityJeddah, Saudi Arabia

**Keywords:** nanostructures, wound healing, chitosan, fibers, polyvivl alcohol

## Abstract

Chitosan is a cheap resource, which is widely used in biomedical applications due to its biocompatible and antibacterial properties. In this study, composite nanofibrous membranes of chitosan (CS) and poly(vinyl alcohol) (PVA) loaded with antibiotics at different ratios were successfully fabricated by electrospinning. The composite nanofibers were subjected to further analysis by scanning electron microscopy (SEM). SEM images revealed that the volumetric ratio of CS/PVA at 50/50 achieved an optimal nanofibrous structure (i.e., that most similar to natural tissues) compared with other volumetric ratios, which indicated that this CS/PVA electrospun scaffold has great potential to be used for infection related wound dressing for skin tissue regeneration.

## Introduction

Chronic dermal wounds, such as infected diabetic foot ulcers, represent a major health problem that affects millions of people worldwide and induces billions of dollars in social and economic costs; the poor treatment outcomes result in high healthcare costs. Such data explain the large research efforts now focused on developing new therapeutic approaches to improve wound healing (Dwivedi et al., 2016). The entire process of normal infection related wound healing requires the formulation of scaffolds with high regeneration properties. Recently electrospinning technology was used as a very popular method to fabricate tissue-engineering scaffolds (Field and Kerstein, 1994; Tchemtchoua et al., 2011). The nanofibrous membrane prepared by electrospinning has its advantages such as high porosity and nanoscale morphology. The electropinning membrane is also important for cell attachment, proliferation, and anti-infection in quick and scarless wound healing. In addition, the wet scaffold plays a very important role in wound healing since water swells in the scaffold and is of great help to reduce necrotic tissue. This is one of the reasons why chitosan is chosen for infection related wound healing because of the rich number of hydrogen bonds between chitosan chains (Homayoni et al., 2009). Previous research revealed that the swelling ratio of chitosan nanofibers is 70% more than chitosan particles (Cooper et al., 2013). Therefore, the objective of this project was to use an electrospinning method to fabricate porous nanofibers loaded with growth factors inside of nanofibers for infection related wound healing improvement.

## Materials and methods

Chitosan solution and poly (vinyl alcohol) (PVA) solution were prepared as described before (Zhou et al., 2013). 3% (w/v) chitosan(Sigma, US) and 8% (w/v) poly (vinyl alcohol) (PVA) (Sigma, US) solutions were optimized and mixed together at different ratios of 80/20, 50/50, and 20/80 (CS/PVA) and were distributed using a sonication method. Ampicillin, one of the most widely used antibiotics, was dissolved in PVA solution with 1% (w/w). The well-distributed CS/PVA solution was then pumped into a plastic syringe separately and electrospinning performed at room temperature. Electrospinning was performed with 17 kV of electric potential applied to a metallic needle using a DC power supply, and the distance between the needle and collector was fixed at 12 cm. The nanofibrous matrix was collected on the surface of aluminum foil and dried at room temperature in vacuum environment overnight. Then, the crosslinking process was carried out in an aqueous glutaraldehyde solution (50%, v/v) (Zhou et al., 2013). The membrane was crosslinked in glutaraldehyde vapor at room temperature for 4 days. After crosslinking, the samples were washed with distilled water and dried in an oven for 24 h. The morphology and diameter of the electrospun samples were observed by scanning electron microscopy (SEM). Mechanical test and antibacterial study will be further investigated in the following studies. All experiments were run in triplicate and repeated three times for each group.

## Results and discussion

**Figure 1:**
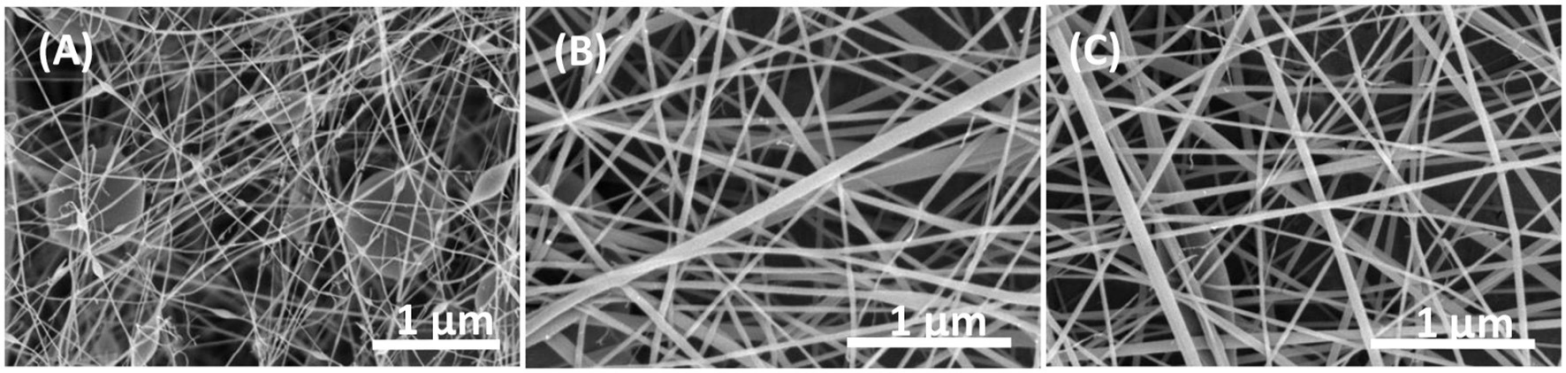
**SEM images of (A)** ratio of 80/20 of CS/PVA, **(B)** ratio of 50/50 of CS/PVA, and **(C)** ratio of 20/80 of CS/PVA.

Chitosan and PVA had been widely investigated for a very long time due to their biocompatible and antibacterial properties. SEM images of nanofibers resulting from different ratio of CS/PVA are shown in Figure 1. It could be found that when the ratio of CS to PVA was equal to 50/50, smooth, and homogeneous fibers were obtained. When the content of chitosan increased, larger fiber diameters were found, which was due to the relatively higher molecule weight of chitosan resulting in a solution hard to electrospun. Besides, numerous beads could be observed based on SEM images (Figure 1A), which also indicated that chitosan with higher molecular weights was hard to electrospun. However, as the ratio of PVA in the mixture increased, the fiber morphology was uneven and very weak as shown in Figure 1C. It might be thought that when a single jet split into multiple filaments because of radical charge repulsion, the polymer solution with high concentrations of PVA could not stand this radical charge repulsion, which resulted in freaking and smaller diameter nanofibers. SEM results indicated that for the CS and PVA at a ratio of 50/50, the nanofibers had optimal nanofibrous structures (i.e., closer to that of natural tissues), making them potential for further study. In addition, both chitosan and antibiotics loaded by PVA has very good effect on antibacterial study based on a previous study (Uygun et al., 2011). All those properties make these CS/PVA/Antibiotics electrospun matrices good wound dressing candidates for infection related wound healing studies.

## Conclusions

In this study, CS/PVA nanofibers were successfully prepared by electrospinning different ratios of CS/PVA solutions. SEM images showed that nanofibers had larger and more nanobeads formed with increasing concentrations of CS, while narrower and breaking nanofibers could be observed if the mixed solution was more than 75% PVA. The results from SEM images showed that the ratio of CS and PVA at 50/50 achieved a nanofibrous structure the most similar to natural tissues. These novel electrospun scaffolds have the potential to be used for infection related wound dressing for skin tissue regeneration.

## Author contributions

MW Performed experiments. AR, Designed and written the paper. TW, Designed and edited.

### Conflict of interest statement

The authors declare that the research was conducted in the absence of any commercial or financial relationships that could be construed as a potential conflict of interest.

## References

[B1] Cooper A. Oldinski R. Ma H. Bryers J. D. Zhang M. (2013). Chitosan-based nanofibrous membranes for antibacterial filter applications. Carbohydr. Polym. 92, 254–259. 10.1016/j.carbpol.2012.08.114 23218292 PMC3579628

[B2] Dwivedi C. Pandey H. Pandey A. C. Ramteke P. W. (2016). Nanofibre based smart pharmaceutical scaffolds for wound repair and regenerations. Curr. Pharm. Des. 22, 1460–1471. 10.2174/1381612822666151215103553 26666999

[B3] Field F. K. Kerstein M. D. (1994). Overview of wound healing in a moist environment. Am. J. Surg. 167, 2S–6S. 8109679 10.1016/0002-9610(94)90002-7

[B4] Homayoni H. Ravandi S. A. H. Valizadeh M. (2009). Electrospinning of chitosan nanofibers: processing optimization. Carbohydr. Polym. 77, 656–661. 10.1016/j.carbpol.2009.02.008

[B5] Tchemtchoua V. T. Atanasova G. Aqil A. Filée P. Garbacki N. Vanhooteghem O.. (2011). Development of a Chitosan Nanofibrillar Scaffold for skin repair and regeneration. Biomacromolecules 12, 3194–3204. 10.1021/bm200680q 21761871

[B7] Uygun A. Kiristi M. Oksuz L. Manolache S. Ulusoy S. (2011). RF hydrazine plasma modification of chitosan for antibacterial activity and nanofiber applications. Carbohydr. Res. 346, 259–265. 10.1016/j.carres.2010.11.020 21159329

[B6] Zhou Y. Yang H. Liu X. Mao J. Gu S. Xu W. (2013). Electrospinning of carboxyethyl chitosan/poly(vinyl alcohol)/silk fibroin nanoparticles for wound dressings. Int. J. Biol. Macromol. 53, 88–92. 10.1016/j.ijbiomac.2012.11.013 23164753

